# New Genetically Manipulated Mice Provide Insights Into the Development and Physiological Functions of Invariant Natural Killer T Cells

**DOI:** 10.3389/fimmu.2018.01294

**Published:** 2018-06-14

**Authors:** Yue Ren, Etsuko Sekine-Kondo, Midori Tateyama, Thitinan Kasetthat, Surasakadi Wongratanacheewin, Hiroshi Watarai

**Affiliations:** ^1^Division of Stem Cell Cellomics, Center for Stem Cell Biology and Regenerative Medicine, Institute of Medical Science, University of Tokyo, Tokyo, Japan; ^2^Department of Neurology, The Neurological Institute of Jiangxi Province, Jiangxi Provincial People’s Hospital, Nanchang, China; ^3^Department of Immunology, Kitasato University School of Medicine, Sagamihara, Japan; ^4^Department of Microbiology, Khon Kaen University, Khon Kaen, Thailand

**Keywords:** invariant natural killer T cells, CD1d, Traj18, iPSC, obesity, adipose tissue, cloned mice, thymic differentiation

## Abstract

Invariant natural killer T (iNKT) cells are a unique T cell subset that exhibits characteristics of both innate immune cells and T cells. They express Vα14-Jα18 (*Trav11*-*Traj18*) as an invariant chain of the T cell receptor (TCR) and are restricted to the MHC class I-like monomorphic antigen presenting molecule CD1d. iNKT cells are known as immune regulators that bridge the innate and acquired immune systems by rapid and massive production of a wide range of cytokines, which could enable them to participate in immune responses during various disease states. Thus, *Traj18*-deficient mice, *Cd1d*-deficient mice, or iNKT cell-overexpressing mice such as iNKT TCRα transgenic mice and iNKT cell cloned mice which contain a Vα14-Jα18 rearrangement in the TCRα locus are useful experimental models for the analysis of iNKT cells *in vivo* and *in vitro*. In this review, we describe the pros and cons of the various available genetically manipulated mice and summarize the insights gained from their study, including the possible roles of iNKT cells in obesity and diabetes.

## Introduction

Invariant natural killer T (iNKT) cells (also called type I NKT cells) are characterized by the expression of an invariant T cell receptor (TCR), Vα14-Jα18 (*Trav11*-*Traj18*) paired with Vβ8.2 (*Trbv13-2*), Vβ8.1 (*Trbv13-3*), Vβ7 (*Trbv29*), or Vβ2 (*Trbv1*) in mice and the Vα24-Jα18/Vβ11-Dβ2-Jβ2.7 (*TRAV10*-*TRAJ18*/*TRBV25-1*-*TRBD2*-*TRBJ2-7*) pair in humans. The iNKT cells differ from classical αβ T cells in recognizing (glyco)lipid antigens (Ags) in conjunction with the monomorphic MHC class I-like CD1d molecule (1, 2). The prototypic Ag recognized by iNKT cells is the glycosphingolipid α-galactosylceramide (α-GalCer), originally isolated from the marine sponge *Agelas mauritianus*. It was identified from structure–activity relationship studies around the glycosphingolipid Agelasphin 9b by the pharmaceutical division of Kirin Brewery Co. Ltd. in a screen for naturally occurring molecules that prevented tumor metastases in mice *in vivo* (3). The synthetic derivative compound, also known as KRN7000 (α-GalCer C26:0), retains the activity of Agelasphin 9b while being much easier to synthesize (4). α-GalCer and its derivatives have been used in many different studies and are highly potent iNKT cell modulators both in humans and in mice (5, 6). Recent studies have demonstrated that iNKT cells, even though they all express the same invariant Ag receptor, can be classified into different functional subtypes, interferon (IFN)-γ-producing iNKT1, interleukin (IL)-13/IL-4-producing iNKT2, and IL-17A-producing iNKT17 (7). When activated by α-GalCer, iNKT cells rapidly produce these various types of cytokines, resulting in bystander immune modulating functions leading to activation and inhibition of various immune effector cells, including NK cells, macrophages, granulocytes, dendritic cells (DCs), basophils, and eosinophils in the innate system as well as CD4^+^ T and CD8^+^ T cells in the acquired system. Therefore, iNKT cells participate in broad spectrum regulation of immune homeostasis and in various disease states including infection, autoimmunity, allergy, antitumor responses, metabolic disorders, allograft rejection, and graft-vs-host disease (8, 9).

Numerous studies investigating the role of iNKT cells have utilized mouse models of iNKT cell deficiency. One such model directly targets Jα18 (*Traj18*^−/−^) (10), which is required for iNKT-TCR formation. However, the overall TCR repertoire diversity is impaired in *Traj18*^−/−^ mice, in which *Traj18* was replaced with a PGK-Neo^r^ cassette, which had inadvertent but substantial effects on transcription and TCRα gene rearrangements (11). Another model makes use of mice deficient in CD1d (*Cd1d1*^−/−^) (12), which prevents the development of any CD1d-restricted T cells including iNKT cells. However, in mice, although not in humans, there has been a gene duplication event and so two homologous genes, *Cd1d1* and *Cd1d2*, encode CD1d proteins. Even though *Cd1d1*^−/−^*Cd1d2*^−/−^ mice have also been developed (13, 14), the role of CD1d2 in iNKT cell development and function is still unclear. Consequently, any changes in immunological activity attributed to iNKT cells that are based on studies of *Traj18*^−/−^ or *Cd1d*^−/−^ mice need to be reassessed.

On the other hand, mice that have been genetically manipulated to have elevated numbers of iNKT cells are also useful tools for iNKT cell study. Therefore, rearranged Vα14-Jα18 and Vβ8 genes were introduced into recombination-activating gene-deficient mice, and there was preferential generation of iNKT cells but no mature B and T lymphocytes (15). iNKT-TCRα transgenic mice that overexpressed iNKT-TCRα (mVα14-Jα18) were firstly generated by Bendelac et al. (16) resulting in preponderance of iNKT cells, while abnormal development of other immune cells were also observed. A human iNKT-TCRα (hVα24-Jα18) transgenic mouse has also been developed by similar approach (17). Moreover, iPS cell lines obtained by reprogramming of mature iNKT cells (iNKT-iPSC) from C57BL/6 (B6) mice preferentially generate iNKT cells but no conventional αβ T or γδ T cells, NK cells, DCs or B cells *in vitro* (18). Furthermore, mice generated from the iNKT-iPSC had a much larger number of iNKT-like cells (19) compared to mice with a rearranged Vα14-Jα18 transgene (16). It is therefore important to compare the development and function of iNKT cells and their subtypes that differentiate *in vivo* in these iNKT cell overexpressed mice.

## *Traj18*-Deficient and *Cd1d*-Deficient Mice

Because iNKT cells are highly conserved among species including mice and humans, mouse models of iNKT cell deficiency represent useful tools for the analysis of iNKT cell biology. However, as described above, the originally generated *Traj18*^−/−^ mice (10) lack transcripts not only of *Traj18* but also of genes encoding Jα regions upstream of *Traj18*, resulting in an almost 60% reduction in the diversity of the TCRα repertoire (11). It is possible that the lower overall αβ TCR diversity of the original *Traj18*^−/−^ mice contributed to the divergent results that have been reported by some of the studies that used the mice. Recently, four new *Traj18*^−/−^ mouse lines have been established by different research groups including ours. Two lines were generated by classical P1 bacteriophage cyclization recombination/locus of crossover in P1 (Cre/loxP) technology (20, 21), a third was generated by transcription activator-like effector nuclease (TALEN) methodology (TALEN-*Traj18*^−/−^) (22), and a fourth by using the clustered regularly interspaced short palindromic repeat (CRISPR)/Cas9 technology (CRISPR-*Traj18*^−/−^) (23). All four groups analyzed TCRα diversity in CD4^+^CD8^+^ double-positive (DP) thymocytes by next-generation sequencing and found that the usage frequency of Jα gene segments in these new *Traj18*^−/−^ mouse lines was comparable with WT B6 mice (20–23).

In addition to canonical Vα14-Jα18 iNKT cells, another minor α-GalCer/CD1d reactive subset of T cells harboring *Trav10*-*Traj50* was recently described as type Ib NKT cells (24). However, type Ib NKT cells were discovered in mice that lack expression of *Traj* gene segments upstream of *Traj18* (10). We (23) and Chandra et al. (20) could not detect any type Ib NKT cells in the new mouse strains lacking iNKT cells. By contrast, Zhang et al. (22) did find type Ib NKT cells in their TALEN-*Traj18*^−/−^ mice. However, these mice express *Trav11*-*Traj18* mRNA with a partial deletion, indicating that a mutant iNKT-TCRα has the unexpected ability to recognize α-GalCer/CD1d. Based on these results, we should rethink the existence of type Ib NKT cells.

**Figure 1:**
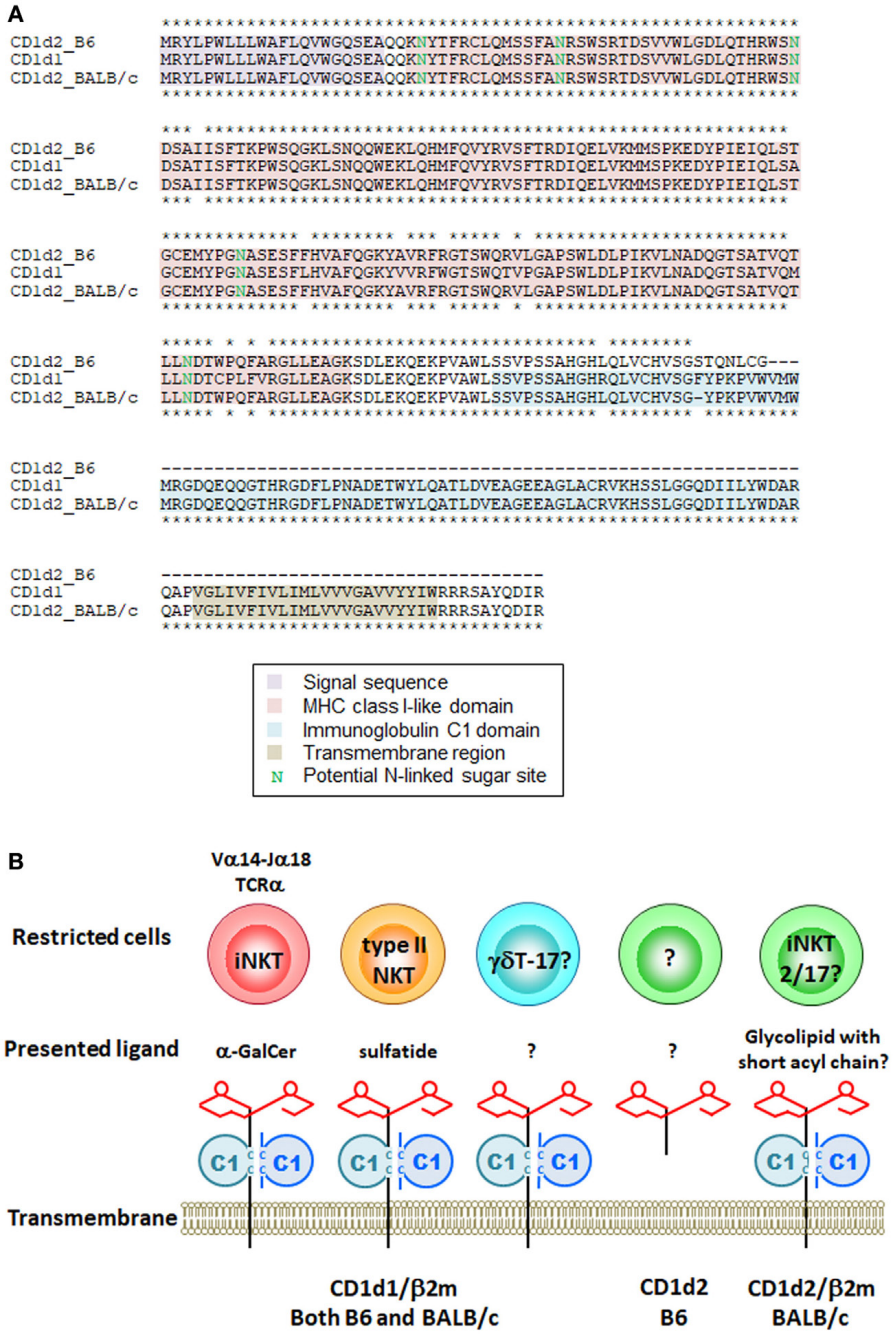
Mouse CD1d molecules and their restricted cells. **(A)** Amino acid sequences of mouse CD1d1 and CD1d2 from B6 and BALB/c mice were aligned by ClustalW. Asterisks indicate identical amino acids between CD1d2 from B6 vs CD1d1 (upper) and CD1d2 from BALB/c vs CD1d1 (lower). **(B)** Schematic representation of mouse CD1d molecules, presented ligands and their restricted cells. The CD1d1 molecule restricts different cells depending on the presented antigen(s). The structure of CD1d2 is different among species in mice due to the frameshift mutation. The CD1d2 molecule in B6 mice has MHC class I-like domain required for the presentation of glycolipid ligand(s) but lacks immunoglobulin C1 domain. It is still unclear whether the soluble form of CD1d2 works as an antigen presenting molecule.

It is known that iNKT cells are restricted by CD1d molecules, but that two CD1d isoforms, CD1d1 and CD1d2, are present in mice. Two gene manipulated lines has been developed, *Cd1d1*^−/−^ (12) and *Cd1d1*^−/−^*Cd1d2*^−/−^ (13, 14). iNKT cells are severely impaired in both lines, indicating that CD1d2 cannot substitute for CD1d1 to support iNKT cell development. CD1d1 and CD1d2 in 129/Sv mice share 93% amino acid identity. Although CD1d2 on thymocytes cannot substitute for the development of iNKT cells (25), we cannot eliminate the potential role of CD1d2 in the development or function of iNKT cells. Sundararaj et al. (26) recently reported that the structure of the CD1d2 A′-pocket was restricted in size compared with CD1d1 in complex with endogenous lipids or a truncated acyl-chain analog of α-GalCer. They found that the majority of iNKT cells in the *Cd1d1*^−/−^ mice showed an increase in the iNKT2 and iNKT17 populations and a concomitant decrease in iNKT1 compared with WT mice (26). A small but consistent increase in the proportion of cells using the Vβ8 gene segment, concomitant to a reduction in Vβ7 gene usage, was also observed for CD1d2-selected iNKT cells compared with CD1d1-selected iNKT cells (26). B6 mice, but not BALB/c or 129/Sv mice, harbor a two-nucleotide insertion in exon 4 of *Cd1d2*, which encodes the α3 domain (27). This frameshift mutation introduces a stop codon, abolishing surface expression but possibly still allowing expression of a soluble CD1d2 molecule (Figure 1A). These results indicate that the CD1d2 molecule can present different sets of self-antigen(s) in the thymus of different mouse strains, thereby potentially impacting the development of iNKT cells. Even though *Cd1d2*^−/−^ mice have not yet been established, they should provide an answer to this controversy.

CD1d is also an Ag presenting molecule for cells other than iNKT cells. Another type of CD1d-restricted cell is the type II or variant NKT cell, which has a more diverse TCR repertoire and appears to recognize various lipid Ags including sulfatides, also known as 3-*O*-sulfogalactosylceramide, which are a class of glycolipids that contain a sulfate group (28). Unfortunately, no tools are yet available that can be used to analyze the entire population of type II NKT cells. Thus, when we discuss type II NKT cells, it is important to understand the advantages and limitations of each experimental tool, as well as the precise definition of type II NKT cells being analyzed (29). There is also a report that the homeostasis of liver-resident IL-17A-producing γδT (γδT-17) cell depends on hepatocyte-expressed CD1d that presents lipid Ag (30); however, it is unclear whether CD1d is required for the development of γδT-17 cells in the thymus (Figure 1B).

Despite a high degree of conservation, subtle but important differences exist between the CD1d Ag presentation pathways of humans and mice. Wen et al. (31) have generated a human CD1d knock-in mouse (hCD1d-KI) which substitute mouse *Cd1d1* to human *CD1D* locus. Reduced numbers of iNKT cells were observed, but at an abundance comparable to that in most humans. They further generated human iNKT-TCRα chain knock-into the hCD1d-KI (32). Similar to humans, the mice developed a subset of CD8αβ^+^ iNKT cells among other human-like iNKT subsets. The results support human *CD1D* is functionally and phenotypically ortholog of mouse *Cd1d1*. These hCD1d-KI mice will allow more accurate *in vivo* modeling of human iNKT cell responses as some human pathogens specifically target human CD1D for pathogenicity and will facilitate the preclinical optimization of iNKT cell-targeted immunotherapies.

## iNKT Cells and Obesity

Obesity research is an illustrative example of how the different genetically engineered animals have been employed to study the role of iNKT cells in a complex disease. Both the original *Traj18*^−/−^ (10) and the *Cd1d*^−/−^ (12–14) mice have been used by many different research groups to study the role of iNKT cells and/or type II NKT cells in obesity-related pathologies in high fat diet (HFD)-induced obesity models. However, these studies have reported very conflicting results, with some groups finding no effect (33, 34), some protection (35–37) and others finding promotion (38, 39) of obesity-associated disease.

Among these studies, only one paper, that published by Lynch et al. (33), has focused on the protective role of iNKT cells in obesity by studying HFD-induced obese *Traj18*^−/−^ mice on a B6 background. In this study, they found that when mice lacking iNKT cells were placed on an HFD they showed enhanced weight gain, larger adipocytes, fatty livers, and insulin resistance. By contrast, many other research groups suggested a pathogenic role of iNKT cells in obesity by showing an ameliorated metabolic phenotype in HFD-induced obese *Traj18*^−/−^ or *Cd1d*^−/−^ mice. In the studies that used B6 background *Traj18*^−/−^ mice, Wu et al. (39) reported ameliorated hepatic steatosis, glucose tolerance, and insulin sensitivity, as well as reduced tissue inflammation in *Traj18*^−/−^ mice on an HFD. Pathological roles of NKT cells in obesity were also reported by Satoh et al. (38) in *Cd1d1*^−/−^ mice; however, no difference in the metabolic parameters between *Traj18*^−/−^ and WT B6 mice on an HFD was observed in their study, arguing for a pathogenic role of type II NKT rather than iNKT cells in these pathologies. Similarly, Kotas et al. (40) and Lee et al. (41) have also reported a minor role of iNKT cells in the development of obesity, by comparing *Traj18*^−/−^ and *Cd1d1*^−/−^*Cd1d2*^−/−^ mice with WT B6 mice on an HFD.

Many reasons for these divergent results have been proposed and discussed, including the age, gender, and background of the mice, HFD type and duration, and the gut flora or environmental microbial distribution among the animals employed by the different research groups. Nevertheless, if we focus only on the results obtained from B6 background *Traj18*^−/−^ mice, it is interesting to note that most of the studies have used an HFD of 60% fat calories, and none of them have reported a decreased level of weight gain in *Traj18*^−/−^ mice as compared with WT B6 mice (36–40). It seems that a consensus has been reached that iNKT cells do not participate in promoting the development of obesity, at least as measured by gain in bodyweight.

To exclude the possible effect of impaired TCR repertoire diversity on diet-induced obesity observed in the original *Traj18*^−/−^ mice (10, 11), we re-investigated the contribution of iNKT cells to the development of obesity using our CRISPR-*Traj18*^−/−^ mice (23) with an unbiased TCR repertoire. The results were clear cut, obese CRISPR-*Traj18*^−/−^ mice gained less body weight and had smaller visceral fat-pads and adipocytes, less fat deposits in the liver, and ameliorated glucose tolerance and insulin resistance (23). The ameliorated levels were almost equivalent to those seen in obese *Cd1d1*^−/−^ mice, indicating that iNKT cells play a pathogenic role in diet-induced obesity and that the impact of CD1d deficiency on metabolism is iNKT cell dependent.

It is notable that one of the T cell populations affected by impaired TCR repertoire diversity in the original *Traj18*^−/−^ mice, the mucosal-associated invariant T (MAIT) cells that use the invariant Vα19-Jα33 (*Trav1-Traj33*) chain in mice (42), were recently reported to have an altered distribution and cytokine productions in obese patients, and were found to be positively associated with insulin resistance (43, 44). Considering the potential role of MAIT cells and other T cell subsets in obesity, results obtained with the original *Traj18*^−/−^ mouse model should be interpreted with caution.

## iNKT-TCRα Transgenic and iNKT Cell Cloned Mice

Mice that have been genetically manipulated to have elevated numbers of iNKT cells were first attempted to generated by overexpressed Vα14-Jα18 iNKT TCRα (mVα14-Jα18) as a transgene (16). Consistent with the results from *Cd1d*-deficient mice (12–14), the mVα14-Jα18 transgenic mice exhibited increased IL-4 and immunoglobulin (Ig) E in serum, indicating that mouse iNKT cells are one of the important sources of IL-4 and IgE. Because both human and mouse iNKT cells are restricted to α-GalCer/CD1d, a human iNKT TCRα (hVα24-Jα18) transgenic mouse has also been developed (17). Interestingly, analysis of the mice and derived hVα24-Jα18^+^ T cells revealed that type 1 diabetes [insulin-dependent diabetes mellitus (IDDM)] is associated with an extreme T helper (Th) 1 phenotype of hVα24-Jα18^+^ T cells, suggesting that there is a strong link between hVα24-Jα18^+^ T cells and human type 1 diabetes. On the other hand, there is evidence that IL-4 exerts a dominant-negative effect on the progression to IDDM in non-obese diabetic (NOD) mice (45–47), and NOD mice with the mVα14-Jα18 transgene were protected from diabetes (48), indicating that not only the number but also the phenotype of iNKT cells influences the incidence of diabetes both in humans and mice. The fact that the gut microbiota can impact iNKT cell development and functions (49–51) and is associated with diabetes onset, regulatory imbalance, and IFN-γ levels in NOD mice should be also considered (52). Another iNKT cell-overexpressing mouse line was derived from iPSCs. iPSCs hold tremendous potential for applications not only in drug discovery, regenerative medicine, and cell replacement therapy (53–55), but also in basic biology. We have succeeded in reprogramming splenic iNKT cells from WT B6 mice (18). These iPSC-iNKT cells could be differentiated into iNKT cells *in vitro* and secreted large amounts of IFN-γ. Importantly, iPSC-iNKT cells recapitulated the known adjuvant effects of natural iNKT cells and suppressed tumor growth *in vivo*. These studies demonstrate the feasibility of expanding functionally competent iNKT cells *via* an iPSC phase, an approach that may be adapted for iNKT cell-targeted therapy in humans (56, 57). We further succeeded in generating iNKT cell cloned *Trav11*-*Traj18^+/+^* mice from iPSC-iNKT cells through germline transmission and breeding with WT B6 mice (19). The absolute numbers and percentages of α-GalCer/CD1d dimer^+^ TCRβ^+^ cells in the thymus and periphery of *Trav11*-*Traj18^+/+^* mice were elevated by 10–20-fold compared to B6 mice and 10–20-fold compared to B6 mice and by 3–10-fold compared to iNKT-TCRα transgenic mice due to the bypass of TCRα rearrangement at the double-negative (DN) stage. They lacked γδ T cells due to the deletion of the δ locus and had reduced numbers of αβ T cells while NK, B, and DC numbers were normal. However, the surface phenotype of α-GalCer/CD1d dimer^+^ TCRβ^+^ cells in *Trav11*-*Traj18^+/+^* mice was different from that in WT B6 mice; there was a partial reduction of CD44^+^ cells and changes in the CD4^+^:NK1.1^+^ ratio (19). We think this is due to the shortage of CD1d molecules in the face of an excess number of α-GalCer/CD1d dimer^+^TCRβ^+^ cells because the surface phenotype of the iNKT cells changed into the mature phenotype as seen in WT B6 when these cells were sorted and transferred into *Traj18*^−/−^ mice (58). Generation of *Trav11*-*Traj18^+/+^* mice carrying a *Cd1d1* transgene should clarify this point.

**Figure 2:**
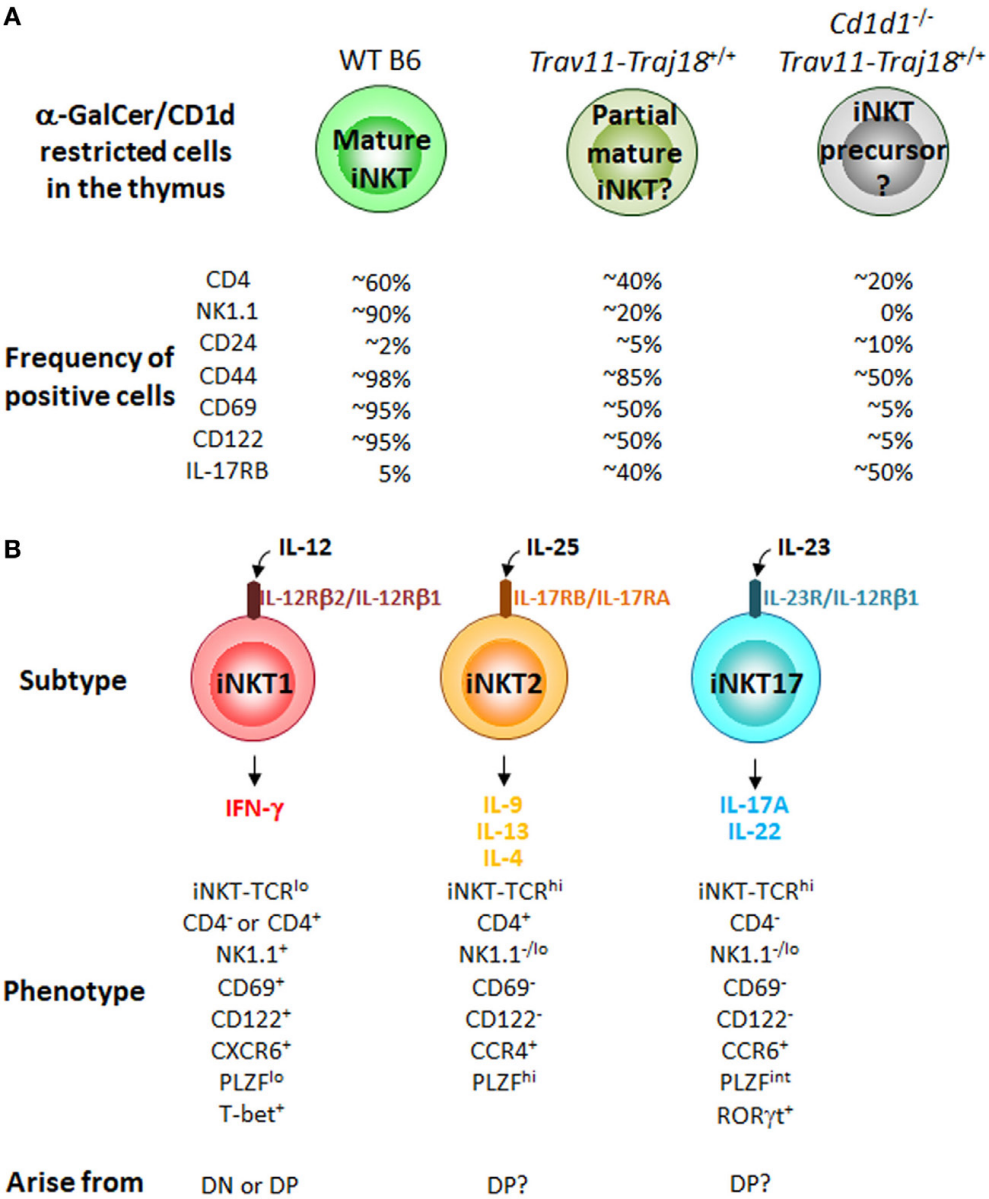
CD1d restricted cells in iPSC-invariant natural killer T (iNKT)-derived cloned mice and iNKT cell subtypes in the thymus of B6 mice. **(A)** Percentage of CD1d-restricted α-GalCer/CD1d dimer^+^TCRβ^+^ cells positive for the indicated cell surface molecules in WT B6, *Trav11*-*Traj18^+/+^* and *Cd1d1*^−/−^*Trav11*-*Traj18^+/+^* mice. **(B)** The iNKT cell subtypes previously characterized in the thymus of B6 mice. Their phenotypes and developmental pathways in the thymus are also shown. Function of iNKT cells is acquired through the development in the thymus distinct from conventional αβ T cells. All of the iNKT subtypes may arise from the CD1d-restricted α-GalCer/CD1d dimer^+^TCRβ^+^ cells in *Cd1d1*^−/−^*Trav11*-*Traj18^+/+^* mice in panel **(A)**, while it still remains to be elucidated which signals control the divergence of iNKT1, iNKT2, and iNKT17 subsets.

*Trav11*-*Traj18^+/+^* mice on a *Cd1d1*^−/−^ background have also been generated (59). Interestingly, these mice have thymic CD1d-restricted α-GalCer/CD1d dimer^+^TCRβ^+^ cells, which are considered to be iNKT cells before CD1d selection. The frequency of positive cells of CD44, CD4, and NK1.1 by thymic α-GalCer/CD1d dimer^+^TCRβ^+^ cells from *Cd1d1*^−/−^*Trav11*-*Traj18^+/+^* mice is further lower than those from *Trav11*-*Traj18^+/+^* mice (Figure 2A), suggesting that CD1d plays a role in the induction of these surface molecules on iNKT cells.

## iNKT Cell Development in the Thymus

Until recently, the iNKT cell field had embraced a sequential lineage developmental model (60) in which “developmental intermediates” produce Th2-type cytokines and “mature” NK1.1^+^ iNKT cells produce Th1 cytokines. However, based on the finding of the expression of distinct transcription factors, T-bet (*Tbx21*), PLZF (*Zbtb16*), and RORγt (*Rorc*) (61) and surface markers, CD4, NK1.1, and IL-17RB (7) in iNKT cell subsets, we considered an alternative “lineage diversification” model for iNKT cells (62), analogous to the differentiation of effector Th cells (63) and innate lymphoid cells (64, 65). Three major subsets of iNKT cells (iNKT1, iNKT2, and iNKT17) that produce distinct cytokines have been defined (7, 61, 66, 67) (Figure 2B), and these represent diverse lineages and not developmental stages, as previously thought. In fact, it was recently reported that some iNKT1 cells developed through an alternative DN pathway that bypasses the DP pathway (68), supporting the above findings that iNKT subtypes possibly arise from different precursors in the thymus. Of note, thymic α-GalCer/CD1d dimer^+^TCRβ^+^ cells from *Cd1d1*^−/−^*Trav11*-*Traj18^+/+^* mice described above exhibit the precursors of all iNKT cell subtypes. The poised effector state is acquired during development in the thymus, where iNKT precursors differentiate into one of three distinct subsets, while it has still been unclear which signals control the divergence of iNKT1, iNKT2, and iNKT17. The precise molecular mechanisms should be clarified that are important for iNKT lineage diversification but are dispensable for conventional αβ T cell development. Taken collectively, it can be proposed that the acquisition of diverse functional characteristics by iNKT subtypes might be dependent on the environment providing an appropriate cytokine milieu, as well as on the cytokine receptor signaling in precursor cells undergoing CD1d selection.

## Conclusion

The gene manipulated mice described here will reveal more insights into mouse iNKT cell development and function, and these insights should also be applicable to human iNKT cell studies. Overall, some reported differences between *Cd1d*^−/−^ and *Traj18*^−/−^ mice are likely due to the loss of some T cell populations including MAIT cells in the original *Traj18*^−/−^ mice. *Traj18*^−/−^ mice with unbiased TCR diversity will inform us of the actual role of iNKT cells and type II NKT cells. *Cd1d1*^−/−^*Trav11*-*Traj18^+/+^* mice may further reveal differences in iNKT cell thymic development and may account for the observed mouse strain specific differences. iNKT cells hold great promise for treatment of a myriad of diseases, and these gene manipulated mice will be invaluable in deciphering the role of iNKT cells in health and disease.

## Author Contributions

All authors listed have made a substantial, direct, and intellectual contribution to the work and approved it for publication.

## Conflict of Interest Statement

The authors declare that the research was conducted in the absence of any commercial or financial relationships that could be construed as a potential conflict of interest.

## References

[1] Godfrey DI MacDonald HR Kronenberg M Smyth MJ Van Kaer L NKT cells: what’s in a name? Nat Rev Immunol (2004) 4:231–7. 10.1038/nri1309 15039760

[2] Salio M Silk JD Jones EY Cerundolo V. Biology of CD1- and MR1-restricted T cells. Annu Rev Immunol (2014) 32:323–66. 10.1146/annurev-immunol-032713-120243 24499274

[3] Natori T Morita M Akimoto K Koezuka Y Agelasphins, novel antitumor and immunostimulatory cerebrosides from the marine sponge *Agelas* mauritianus. Tetrahedron (1994) 50:2771–84. 10.1016/S0040-4020(01)86991-X

[4] Morita M Motoki K Akimoto K Natori T Sakai T Sawa E Structure-activity relationship of alpha-galactosylceramides against B16-bearing mice. J Med Chem (1995) 38:2176–87. 10.1021/jm00012a018 7783149

[5] Banchet-Cadeddu A Hénon E Dauchez M Renault JH Monneaux F Haudrechy A. The stimulating adventure of KRN7000. Org Biomol Chem (2011) 9:3080–104. 10.1039/c0ob00975j 21394364

[6] Laurent X Bertin B Renault N Farce A Speca S Milhomme O Switching invariant natural killer T (iNKT) cell response from anticancerous to anti-inflammatory effect: molecular bases. J Med Chem (2014) 57:5489–508. 10.1021/jm4010863 24428717

[7] Watarai H Sekine-Kondo E Shigeura T Motomura Y Yasuda T Satoh R Development and function of invariant natural killer T cells producing Th2- and Th17-cytokines. PLoS Biol (2012) 10:e1001255 10.1371/journal.pbio.1001255 22346732 PMC3274505

[8] Taniguchi M Harada M Kojo S Nakayama T Wakao H. The regulatory role of Valpha14 NKT cells in innate and acquired immune response. Annu Rev Immunol (2003) 21:483–513. 10.1146/annurev.immunol.21.120601.141057 12543936

[9] Bendelac A Savage PB Teyton L. The biology of NKT cells. Annu Rev Immunol (2007) 25:297–336. 10.1146/annurev.immunol.25.022106.141711 17150027

[10] Cui J Shin T Kawano T Sato H Kondo E Toura I Requirement for Valpha14 NKT cells in IL-12-mediated rejection of tumors. Science (1997) 278:1623–6. 10.1126/science.278.5343.1623 9374462

[11] Bedel R Matsuda JL Brigl M White J Kappler J Marrack P Lower TCR repertoire diversity in Traj18-deficient mice. Nat Immunol (2013) 13:705–6. 10.1038/ni.2347 PMC3748587 22814339

[12] Mendiratta SK Martin WD Hong S Boesteanu A Joyce S Van Kaer L. CD1d1 mutant mice are deficient in natural T cells that promptly produce IL-4. Immunity (1997) 6:469–77. 10.1016/S1074-7613(00)80290-3 9133426

[13] Smiley ST Kaplan MH Grusby MJ. Immunoglobulin E production in the absence of interleukin-4-secreting CD1-dependent cells. Science (1997) 275:977–9. 10.1126/science.275.5302.977 9020080

[14] Chen YH Chiu NM Mandal M Wang N Wang CR. Impaired NK1^+^ T cell development and early IL-4 production in CD1-deficient mice. Immunity (1997) 6:459–67. 10.1016/S1074-7613(00)80289-7 9133425

[15] Kawano T Cui J Koezuka Y Toura I Kaneko Y Motoki K CD1d-restricted and TCR-mediated activation of valpha14 NKT cells by glycosylceramides. Science (1997) 278:1626–9. 10.1126/science.278.5343.1626 9374463

[16] Bendelac A Hunziker RD Lantz O. Increased interleukin 4 and immunoglobulin E production in transgenic mice overexpressing NK1 T cells. J Exp Med (1996) 184:1285–93. 10.1084/jem.184.4.1285 8879200 PMC2192838

[17] Wilson SB Kent SC Patton KT Orban T Jackson RA Exley M Extreme Th1 bias of invariant Valpha24JalphaQ T cells in type 1 diabetes. Nature (1998) 391:177–81. 10.1038/34419 9428763

[18] Watarai H Fujii S Yamada D Rybouchkin A Sakata S Nagata Y Murine induced pluripotent stem cells can be derived from and differentiate into natural killer T cells. J Clin Invest (2010) 120:2610–8. 10.1172/JCI42027 20516640 PMC2898602

[19] Ren Y Dashtsoodol N Watarai H Koseki H Quan C Taniguchi M. Generation of induced pluripotent stem cell-derived mice by reprogramming of a mature NKT cell. Int Immunol (2014) 26:551–61. 10.1093/intimm/dxu057 24854340 PMC4169672

[20] Chandra S Zhao M Budelsky A de Mingo Pulido A Day J Fu Z A new mouse strain for the analysis of invariant NKT cell function. Nat Immunol (2015) 16:799–800. 10.1038/ni.3203 26075912 PMC4711267

[21] Dashtsoodol N Shigeura T Ozawa R Harada M Kojo S Watanabe T Generation of novel Traj18-deficient mice lacking Vα14 natural killer T cells with an undisturbed T cell receptor α-chain repertoire. PLoS One (2016) 11:e0153347 10.1371/journal.pone.0153347 27064277 PMC4827811

[22] Zhang J Bedel R Krovi SH Tuttle KD Zhang B Gross J Mutation of the Traj18 gene segment using TALENs to generate natural killer T cell deficient mice. Sci Rep (2016) 6:27375. 10.1038/srep27375 27256918 PMC4891675

[23] Ren Y Sekine-Kondo E Shibata R Kato-Itoh M Umino A Yanagida A A novel mouse model of iNKT cell-deficiency generated by CRISPR/Cas9 reveals a pathogenic role of iNKT cells in metabolic disease. Sci Rep (2017) 7:12765. 10.1038/s41598-017-12475-4 28986544 PMC5630609

[24] Uldrich AP Patel O Cameron G Pellicci DG Day EB Sullivan LC A semi-invariant Vα10^+^ T cell antigen receptor defines a population of natural killer T cells with distinct glycolipid antigen-recognition properties. Nat Immunol (2011) 12:616–23. 10.1038/ni.2051 21666690 PMC5584938

[25] Chen YH Wang B Chun T Zhao L Cardell S Behar SM Expression of CD1d2 on thymocytes is not sufficient for the development of NK T cells in CD1d1-deficient mice. J Immunol (1999) 162:4560–6. 10201995

[26] Sundararaj S Zhang J Krovi SH Bedel R Tuttle KD Veerapen N Differing roles of CD1d2 and CD1d1 proteins in type I natural killer T cell development and function. Proc Natl Acad Sci U S A (2018) 115:E1204–13. 10.1073/pnas.1716669115 29351991 PMC5819427

[27] Park SH Roark JH Bendelac A. Tissue-specific recognition of mouse CD1 molecules. J Immunol (1998) 160:3128–34. 9531267

[28] Halder RC Aguilera C Maricic I Kumar V. Type II NKT cell-mediated anergy induction in type I NKT cells prevents inflammatory liver disease. J Clin Invest (2007) 117:2302–12. 10.1172/JCI31602 17641782 PMC1913490

[29] Kato S Berzofsky JA Terabe M. Possible therapeutic application of targeting type II natural killer T cell-mediated suppression of tumor immunity. Front Immunol (2018) 9:314. 10.3389/fimmu.2018.00314 29520281 PMC5827362

[30] Li F Hao X Chen Y Bai L Gao X Lian Z The microbiota maintain homeostasis of liver resident γδT-17 cells in a lipid antigen/CD1d dependent manner. Nat Commun (2017) 8:13839 10.1038/ncomms13839 PMC5227332 28067223

[31] Wen X Rao P Carreño LJ Kim S Lawrenczyk A Porcelli SA Human CD1d knock-in mouse model demonstrates potent antitumor potential of human CD1d-restricted invariant natural killer T cells. Proc Natl Acad Sci U S A (2013) 110:2963–8. 10.1073/pnas.1300200110 23382238 PMC3581944

[32] Wen X Kim S Xiong R Li M Lawrenczyk A Huang X A subset of CD8αβ^+^ invariant NKT cells in a humanized mouse model. J Immunol (2015) 195:1459–69. 10.4049/jimmunol.1500574 26157173 PMC4530047

[33] Mantell BS Stefanovic-Racic M Yang X Dedousis N Sipula IJ O’Doherty RM Mice lacking NKT cells but with a complete complement of CD8^+^ T cells are not protected against the metabolic abnormalities of diet-induced obesity. PLoS One (2011) 6:e19831 10.1371/journal.pone.0019831 21674035 PMC3108591

[34] Subramanian S Goodspeed L Wang S Ding Y O’Brien KD Getz GS Deficiency of invariant natural killer T cells does not protect against obesity but exacerbates atherosclerosis in *Ldlr*^-/-^ mice. Int J Mol Sci (2018) 19:E510. 10.3390/ijms19020510 29419749 PMC5855732

[35] Lynch L Nowak M Varghese B Clark J Hogan AE Toxavidis V Adipose tissue invariant NKT cells protect against diet-induced obesity and metabolic disorder through regulatory cytokine production. Immunity (2012) 37:574–87. 10.1016/j.immuni.2012.06.016 22981538 PMC4991771

[36] Schipper HS Rakhshandehroo M van de Graaf SF Venken K Koppen A Stienstra R Natural killer T cells in adipose tissue prevent insulin resistance. J Clin Invest (2012) 122:3343–54. 10.1172/JCI62739 22863618 PMC3428087

[37] Ji Y Sun S Xu A Bhargava P Yang L Lam KS Activation of natural killer T cells promotes M2 macrophage polarization in adipose tissue and improves systemic glucose tolerance via interleukin-4 (IL-4)/STAT6 protein signaling axis in obesity. J Biol Chem (2012) 287:13561–71. 10.1074/jbc.M112.350066 22396530 PMC3340139

[38] Satoh M Andoh Y Clingan CS Ogura H Fujii S Eshima K Type II NKT cells stimulate diet-induced obesity by mediating adipose tissue inflammation, steatohepatitis and insulin resistance. PLoS One (2012) 7:e30568. 10.1371/journal.pone.0030568 22383967 PMC3284453

[39] Wu L Parekh VV Gabriel CL Bracy DP Marks-Shulman PA Tamboli RA Activation of invariant natural killer T cells by lipid excess promotes tissue inflammation, insulin resistance, and hepatic steatosis in obese mice. Proc Natl Acad Sci U S A (2012) 109:E1143–52. 10.1073/pnas.1200498109 22493234 PMC3358828

[40] Kotas ME Lee HY Gillum MP Annicelli C Guigni BA Shulman GI Impact of CD1d deficiency on metabolism. PLoS One (2011) 6:e25478. 10.1371/journal.pone.0025478 21980475 PMC3183002

[41] Lee BC Lee J. Cellular and molecular players in adipose tissue inflammation in the development of obesity-induced insulin resistance. Biochim Biophys Acta (2014) 1842:446–62. 10.1016/j.bbadis.2013.05.017 23707515 PMC3800253

[42] Reantragoon R Corbett AJ Sakala IG Gherardin NA Furness JB Chen Z Antigen-loaded MR1 tetramers define T cell receptor heterogeneity in mucosal-associated invariant T cells. J Exp Med (2013) 210:2305–20. 10.1084/jem.20130958 24101382 PMC3804952

[43] Carolan E Tobin LM Mangan BA Corrigan M Gaoatswe G Byrne G Altered distribution and increased IL-17 production by mucosal-associated invariant T cells in adult and childhood obesity. J Immunol (2015) 194:5775–80. 10.4049/jimmunol.1402945 25980010

[44] Magalhaes I Pingris K Poitou C Bessoles S Venteclef N Kiaf B Mucosal-associated invariant T cell alterations in obese and type 2 diabetic patients. J Clin Invest (2015) 125:1752–62. 10.1172/JCI78941 25751065 PMC4396481

[45] Rapoport MJ Jaramillo A Zipris D Lazarus AH Serreze DV Leiter EH Interleukin 4 reverses T cell proliferative unresponsiveness and prevents the onset of diabetes in nonobese diabetic mice. J Exp Med (1993) 178:87–99. 10.1084/jem.178.1.87 8315397 PMC2191073

[46] Mueller R Krahl T Sarvetnick N. Pancreatic expression of interleukin-4 abrogates insulitis and autoimmune diabetes in nonobese diabetic (NOD) mice. J Exp Med (1996) 184:1093–9. 10.1084/jem.184.3.1093 9064326 PMC2192796

[47] Delovitch TL Singh B The nonobese diabetic mouse as a model of autoimmune diabetes: immune dysregulation gets the NOD. Immunity (1997) 7:727–38. 10.1016/S1074-7613(00)80392-1 9430219

[48] Lehuen A Lantz O Beaudoin L Laloux V Carnaud C Bendelac A Overexpression of natural killer T cells protects Vα14-Jα281 transgenic nonobese diabetic mice against diabetes. J Exp Med (1998) 188:1831–9. 10.1084/jem.188.10.1831 9815260 PMC2212408

[49] Wei B Wingender G Fujiwara D Chen DY McPherson M Brewer S Commensal microbiota and CD8^+^ T cells shape the formation of invariant NKT cells. J Immunol (2010) 184:1218–26. 10.4049/jimmunol.0902620 20048124 PMC3458428

[50] Olszak T An D Zeissig S Vera MP Richter J Franke A Microbial exposure during early life has persistent effects on natural killer T cell function. Science (2012) 336:489–93. 10.1126/science.1219328 22442383 PMC3437652

[51] Wingender G Stepniak D Krebs P Lin L McBride S Wei B Intestinal microbes affect phenotypes and functions of invariant natural killer T cells in mice. Gastroenterology (2012) 143:418–28. 10.1053/j.gastro.2012.04.017 22522092 PMC3404247

[52] Krych Ł Nielsen DS Hansen AK Hansen CH Gut microbial markers are associated with diabetes onset, regulatory imbalance, and IFN-γ level in NOD mice. Gut Microbes (2015) 6:101–9. 10.1080/19490976.2015.1011876 25648687 PMC4615729

[53] Takahashi K Yamanaka S. Induction of pluripotent stem cells from mouse embryonic and adult fibroblast cultures by defined factors. Cell (2006) 126:663–76. 10.1016/j.cell.2006.07.024 16904174

[54] Takahashi K Tanabe K Ohnuki M Narita M Ichisaka T Tomoda K Induction of pluripotent stem cells from adult human fibroblasts by defined factors. Cell (2007) 131:861–72. 10.1016/j.cell.2007.11.019 18035408

[55] Shi Y Inoue H Wu JC Yamanaka S. Induced pluripotent stem cell technology: a decade of progress. Nat Rev Drug Discov (2017) 16:115–30. 10.1038/nrd.2016.245 27980341 PMC6416143

[56] Kitayama S Zhang R Liu TY Ueda N Iriguchi S Yasui Y Cellular adjuvant properties, direct cytotoxicity of re-differentiated Vα24 invariant NKT-like cells from human induced pluripotent stem cells. Stem Cell Reports (2016) 6:213–27. 10.1016/j.stemcr.2016.01.005 26862702 PMC4750166

[57] Yamada D Iyoda T Vizcardo R Shimizu K Sato Y Endo TA Efficient regeneration of human Vα24+ invariant natural killer T cells and their anti-tumor activity *in vivo*. Stem Cells (2016) 34:2852–60. 10.1002/stem.2465 27422351

[58] Watarai H Rybouchkin A Hongo N Nagata Y Sakata S Sekine E Generation of functional NKT cells *in vitro* from embryonic stem cells bearing rearranged invariant Vα14-Jα18 TCRα gene. Blood (2010) 115:230–7. 10.1182/blood-2009-04-217729 19897575

[59] Wakao H Kawamoto H Sakata S Inoue K Ogura A Wakao R A novel mouse model for invariant NKT cell study. J Immunol (2007) 179:3888–95. 10.4049/jimmunol.179.6.3888 17785826

[60] Benlagha K Kyin T Beavis A Teyton L Bendelac A. A thymic precursor to the NK T cell lineage. Science (2002) 296:553–5. 10.1126/science.1069017 11968185

[61] Lee YJ Holzapfel KL Zhu J Jameson SC Hogquist KA. Steady-state production of IL-4 modulates immunity in mouse strains and is determined by lineage diversity of iNKT cells. Nat Immunol (2013) 14:1146–54. 10.1038/ni.2731 24097110 PMC3824254

[62] Constantinides MG Bendelac A. Transcriptional regulation of the NKT cell lineage. Curr Opin Immunol (2013) 25:161–7. 10.1016/j.coi.2013.01.003 23402834 PMC3647452

[63] Zhu J Yamane H Paul WE Differentiation of effector CD4 T cell populations. Annu Rev Immunol (2010) 28:445–89. 10.1146/annurev-immunol-030409-101212 20192806 PMC3502616

[64] Spits H Artis D Colonna M Diefenbach A Di Santo JP Eberl G Innate lymphoid cells – a proposal for uniform nomenclature. Nat Rev Immunol (2013) 13:145–9. 10.1038/nri3365 23348417

[65] Ishizuka IE Constantinides MG Gudjonson H Bendelac A. The innate lymphoid cell precursor. Annu Rev Immunol (2016) 34:299–316. 10.1146/annurev-immunol-041015-055549 27168240

[66] Terashima A Watarai H Inoue S Sekine E Nakagawa R Hase K A novel subset of mouse NKT cells bearing the IL-17 receptor B responds to IL-25 and contributes to airway hyperreactivity. J Exp Med (2008) 205:2727–33. 10.1084/jem.20080698 19015310 PMC2585837

[67] Michel ML Mendes-da-Cruz D Keller AC Lochner M Schneider E Dy M Critical role of ROR-γt in a new thymic pathway leading to IL-17-producing invariant NKT cell differentiation. Proc Natl Acad Sci U S A (2008) 105:19845–50. 10.1073/pnas.0806472105 19057011 PMC2604995

[68] Dashtsoodol N Shigeura T Aihara M Ozawa R Kojo S Harada M Alternative pathway for the development of Vα14 + NKT cells directly from CD4^-^CD8^-^ thymocytes that bypasses the CD4^+^CD8^+^ stage. Nat Immunol (2017) 18:274–82. 10.1038/ni.3668 28135253

